# House Dust Mite Exposure Causes Increased Susceptibility of Nasal Epithelial Cells to Adenovirus Infection

**DOI:** 10.3390/v12101151

**Published:** 2020-10-11

**Authors:** Malik Aydin, Ella A. Naumova, Friedrich Paulsen, Wenli Zhang, Felix Gopon, Christian Theis, Sören Lutz, Eric Ehrke-Schulz, Wolfgang H. Arnold, Stefan Wirth, Anja Ehrhardt

**Affiliations:** 1 Children’s Hospital, Center for Clinical and Translational Research (CCTR), Helios University Medical Center Wuppertal, Witten/Herdecke University, 42283 Wuppertal, Germany; malik.aydin@uni-wh.de (M.A.); stefan.wirth@uni-wh.de (S.W.); 2 Laboratory of Clinical Molecular Genetics & Epigenetics, Center for Biomedical Education and Research, School of Life Sciences (ZBAF), Faculty of Health, Witten/Herdecke University, 42283 Wuppertal, Germany; 3 Department of Biological and Material Sciences in Dentistry, Faculty of Health, Witten/Herdecke University, 58455 Witten, Germany; ella.naumova@uni-wh.de (E.A.N.); wolfgang.arnold@uni-wh.de (W.H.A.); 4 Institute of Functional and Clinical Anatomy, Friedrich Alexander University Erlangen-Nürnberg, 91054 Erlangen, Germany; friedrich.paulsen@fau.de; 5 Department of Topographic Anatomy and Operative Surgery, Sechenov University, 119146 Moscow, Russia; 6 Institute of Virology and Microbiology, Center for Biomedical Education and Research (ZBAF), Department of Human Medicine, Faculty of Health, Witten/Herdecke University, 58453 Witten, Germany; wenli.zhang@uni-wh.de (W.Z.); eric.ehrke-schulz@uni-wh.de (E.E.-S.); 7 Clinics for Anesthesiology, Helios University Medical Center Wuppertal, Center for Clinical and Translational Research (CCTR), Witten/Herdecke University, 42283 Wuppertal, Germany; felix.gopon@helios-gesundheit.de (F.G.); christian.theis@helios-gesundheit.de (C.T.); 8 Children’s Hospital, Helios Hospital Niederberg, Teaching Hospital of University Hospital Essen, 42549 Velbert, German; soeren.lutz@helios-gesundheit.de

**Keywords:** adenovirus, house dust mite, CAR, CD46, allergy, asthma, pathogenesis

## Abstract

Adenovirus (AdV) infections in the respiratory tract may cause asthma exacerbation and allergic predisposition, and the house dust mite (HDM) may aggravate virus-induced asthma exacerbations. However, the underlying mechanisms of whether and how AdV affects asthmatic patients remains unclear. To address this question, we investigated nasal epithelial cells (NAEPCs) derived from a pediatric exacerbation study cohort for experimental analyses. We analyzed twenty-one different green-fluorescent protein- and luciferase-tagged AdV types in submerged 2D and organotypic 3D cell culture models. Transduction experiments revealed robust transduction of AdV type 5 (AdV5) in NAEPCs, which was associated with an increased uptake of AdV5 in the presence of HDM. In healthy and asthmatic NAEPCs exposed to HDM before infection, we observed a time- and dose-dependent increase of AdV5 uptake associated with upregulation of entry receptors for AdV5. Furthermore, electron microscopic and histologic analyses of 3D cell cultures revealed an impairment of the respiratory cilia after HDM exposition. This ex vivo pilot study shows the impact of AdV infection and HDM exposition in a primary cell culture model for asthma.

## 1. Introduction

Currently, more than 100 human adenoviruses (AdV) (http://hadvwg.gmu.edu/) have been identified. They have been phylogenetically divided into seven species (A to G) based on hemagglutination features, oncogenic potential in rodents, DNA homology, and genome organization [1,2]. Adenoviruses are non-enveloped viruses, which contain a double-stranded DNA viral genome of an approximate length of 26–46 kbp [3]. The capsid consists of 252 capsomeres, and the virus shape is icosahedral with 240 hexon-, 12 penton-, and fiber proteins including shaft and knob [4]. For AdV cell entry, several cellular receptors have been described, including the coxsackie andadenovirus receptor (CAR), CD46, sialic acid, desmoglein-2 (DSG-2), and heparan sulfate proteoglycan (HSPG) [4]. AdV are known as pathogens, but they have also been explored as viral vectors in gene therapeutic applications. In clinics, human AdV have become increasingly important in recent years. They cause different clinical symptoms in a wide range of diseases, e.g., pneumonia, conjunctivitis, gastroenteritis, or myocarditis [5,6,7,8]. Threatened groups include children younger than five years of age or immune-deficient patients after transplantation. In addition, AdV have also been causatively associated with pneumonia outbreaks in US-military bases [9]. Several AdV can be isolated from patients with lung infections [8], and here we addressed the question of whether this is associated with asthma exacerbation.

There is strong evidence that asthma exacerbations are associated with virus-mediated upper and/or lower respiratory infections [10], and therefore there is a broad interest in studying the role of viruses in asthma pathogenesis and exacerbation [11,12,13,14]. It was described that predominantly rhinovirus (RV), and other viruses, particularly adenoviruses (AdV), cytomegalovirus, bocavirus, coronavirus, herpes simplex virus, influenza virus, parainfluenza virus, respiratory syncytial virus, or enteroviruses, may be involved in asthma development [15]. In addition to viruses, house dust mite (HDM) as a major allergen is strongly associated with asthma and presents an important risk factor for virus-induced asthma exacerbation [12,13]. Although various studies have investigated the molecular roles of some respiratory viruses in allergic pathways [14,15], the relationship between AdV infection and HDM sensitization in asthma exacerbation has not been sufficiently analyzed. Here, we aimed at analyzing this relationship in primary nasal epithelial cells (NAEPCs) as an ex vivo cell culture model, to better study allergies and the immunology of asthma [16,17,18].

To analyze AdV infection in the context of HDM sensitization, we utilized primary nasal NAEPCs derived from our pediatric exacerbation study cohort in submerged 2D and organotypic 3D cell culture models. For this approach, we applied twenty-one previously described green-fluorescent protein (GFP)- and luciferase-tagged AdV types [19], encompassing AdV of all seven species. Moreover, we performed electron microscopic and histologic analyses of 3D cell cultures to study the impairment of respiratory cilia after HDM exposure. We found that HDM exposure may increase AdV5 infection in vitro, and that major AdV surface receptors may play a role.

## 2. Materials and Methods

### 2.1. Subjects and Study Design

We established a pediatric exacerbation study network in two children’s hospitals in Germany. Participating study centers were Wuppertal (Witten/Herdecke University, Germany) and Niederberg/Velbert (teaching hospital of the University Hospital Essen, Germany). Pediatric subjects with a chronic bronchitis/wheeze (3 months to ≤5 years of age) or asthma (>6 years to 17 years of age) suffering from acute exacerbation episodes were recruited. In addition, healthy controls (age range >3 months to 65 years of age) were recruited for comparison. A detailed study description and protocol is currently in preparation for publication. For the experimental approach used here, only NAEPCs from asthmatics and healthy donors were used (*n* = 3 per each group as biologic with each *n* = 3 technical replicates). For the control experiments, cells from healthy donors were compared with cells from asthmatic subjects (treated and untreated). Only asthmatics with an HDM sensitization (released IgE levels in serum, positive ImmunoCAP^®^ results for *Dermatophagoides pteronyssinus*, CAP class >3) were chosen for this work. Furthermore, only healthy donors without any positive sensitization to *Dermatophagoides pteronyssinus* were selected. Clinically, asthmatic patients showed an immunologic reaction to *Dermatophagoides pteryonossinus,* and the healthy donors did not present any allergic reactions. For this prospective study, all analyzed biomaterials and data involving human participants were collected in concordance with the ethical standards and with the 1964 Helsinki declaration and its later amendments or comparable ethical standards. Ethics approval was obtained from the local Ethics Committees (Witten/Herdecke University (158/2017) and Medical chamber (Ärztekammer Nordrhein, NR 2019312), Germany). The study was retrospectively assigned the human study at German Clinical Trials Register (DRKS) with the registration number DRKS00015738. All study cohort relevant data analyses were pseudonymously performed. All participated subjects or their legal custodians/parents provided a written informed consent. This article does not contain any animal studies.

### 2.2. Technical Information

#### 2.2.1. Culturing of NAEPCs in 2D and 3D Models

The NAEPCs were obtained by performing a nasal brushing (Cytobrush eSwab^®^ Copan Italia) from both nostrils, resuspended in warm BEGM^®^ medium (purchased from Lonza, Basel, Switzerland), and shaken for 30 s. Approximately, 150,000–250,000 cells per nasal brushing procedure (including both nostrils) were collected, the total number was approximately 1 × 10^6^ cells at passage 2 (P2). To eliminate possible contamination with erythrocytes and fibroblasts, lysing procedures were performed before experimental set-up. After centrifugation at 350× *g* for 8 min, the cell number was calculated using a Neubauer counting chamber. Cells were incubated in collagen I pre-coated T75 flasks (Greiner Bio-One, Austria) for up to a maximum of two passages (P2) at 37 °C and 5% CO_2_ atmosphere. At P2, the cells were seeded using an organotypic 3D air liquid interface (ALI) cell culturing technique adapted to the instructions of STEMCELL^TM^ Technologies (https://www.stemcell.com/). For this, 25,000 cells were seeded in collagen I pre-coated transwells using PneumaCult^TM^ ALI proliferation medium added to the basal and apical chambers, and the medium was changed every single day. At day 4, the medium in both chambers were removed, and the cells received PneumaCult^TM^ ALI differentiation medium at the basal chamber. The medium was changed every 2 to 3 days assuring airlifting for up to 4 weeks until the pseudostratified morphology was reached, which was confirmed by ciliary beats and production of mucus (PneumaCult^TM^ ALI and differentiation medium were purchased from STEMCELL^TM^ Technologies, Canada).

#### 2.2.2. Flow Cytometric Characterization of NAEPCs and Adenoviral Receptors

NAEPCs were characterized through flow cytometry using CD45-APC (Miltenyi Biotec, Germany), CD326 (EpCAM)-PE (Miltenyi Biotec, Germany), and anti-cytokeratin-FITC (Miltenyi Biotec, Germany), followed by fixation and permeabilization using Inside Stain Kit (Miltenyi Biotec, Germany) according to manufacturer’s instructions. The selected antibodies for the characterization of NAEPCs were adapted in a previously published study [20]. The AdV receptors, CD46-APC (Miltenyi Biotec, Germany), CXADR/CAR-PE, and DSG-2-PE (Affymetrix, Thermo Fisher Scientific) were chosen for flow cytometry analyses.

#### 2.2.3. Scanning Electron Microscopic Imaging of Organotypic 3D NAEPCs Cultures

The specimens were fixed with 0.1 M cacodylate buffer containing 2.5% glutaraldehyde, 2% polyvinylpyrrolidone, and 75 mM NaNO_2_ for 30 min. at 4 °C. Samples were washed in 0.1 M cacodylate buffer without glutaraldehyde and subsequently incubated in a solution containing 2% arginine-HCl, glycine, sucrose, and sodium glutamate for 18 h at room temperature (RT). The specimens were rinsed in distilled water, followed by immersion in a mixture of each 2% tannic acid and guanidine-HCl for 6 h at RT. The samples were rinsed again in 0.1 M cacodylate buffer and incubated in a 1% OsO_4_ solution for 30 min at RT. After three rinsing steps with 0.1 M cacodylate buffer, the specimens were dehydrated, dried in liquid CO_2_, and finally examined with a Zeiss Sigma SEM (Zeiss, Oberkochen, Germany) scanning electron microscope using 2 kV acceleration voltage after sputtering with gold palladium. As detectors, the in-lens and SE detectors were used.

#### 2.2.4. Transmission Electron Microscopic (TEM) Imaging of Organotypic 3D NAEPCs Cultures

The specimens were processed for TEM according to a previously published protocol [21]. In brief, samples were fixed in Ito’s fixative (2.5% glutaraldehyde, 2.5% paraformaldehyde, and 0.3% picric acid) dissolved in phosphate buffered saline (PBS) (pH = 7.3) and embedded in Epon. Semi-thin sagittal sections of 1 μm were cut with a microtome (Ultracut E; Reichert Jung, Vienna, Austria) and subsequently stained with toluidine blue. Sections were viewed with an epifluorescence microscope (Aristoplan; Ernst Leitz, Wetzlar, Germany) and photographed (Keyence Biorevo BZ9000 microscope). Ultrathin sections were stained with uranyl acetate and lead citrate and viewed with a transmission electron microscope (EM109; Carl Zeiss Meditec GmbH, Oberkochen, Germany).

#### 2.2.5. House Dust Mite Induced Exposition In Vitro

The NAEPCs were seeded in collagen coated 96 wells (day 0). At day 1, the culture medium was changed. For exposition experiments, *Dermatophagoides pteronyssinus* (CITEQ Biologics, Netherlands, product code: 02.01.64) was used in different concentrations (1 µg/mL, 10 µg/mL, and 100 µg/mL) for two time points. The first time point included an exposition duration of 24 h. The read-out parameters will be presented in the course of the manuscript. After 24 h exposition with *Dermatophagoides pteronyssinus*, the supernatant was removed, fresh BEGM^®^ cell culture medium was added, and NAEPCs were incubated for 24 h. After this incubation time, a second exposition with *Dermatophagoides pteronyssinus* was performed for 24 h, and after that, the read-out parameters were measured (see below). These two time points were presented as 24 h and 72 h throughout the manuscript. In addition, to easily follow the manuscript, the term HDM was used for *Dermatophagoides pteronyssinus* throughout the manuscript.

#### 2.2.6. Vector Production and Titration

We used different recombinant AdV types deleted for the early gene region E3, which was replaced by a transgene expression cassette encoding the reporter genes luciferase and GFP [19]. In brief, recombinant viruses were amplified in permissive cell lines and purified using cesium-chloride gradients as described before [19]. We explored *n* = 21 different AdV types derived from different species to transduce NAEPCs. Twenty-four hours before transduction, we seeded 1 × 10^4^ NAEPCs per well in collagen I pre-coated 96 wells plates. GFP and luciferase expressing AdV types were added to each well using 50 viral particles per cell (vpc). Twenty-four hours post transduction of NAEPCs, we determined luciferase activity within the transduced cells using the Nano-Glo^®^ Luciferase Assay System from Promega. To perform the luciferase assay, we removed 100 µl supernatant, added Nano-Glo^®^ Luciferase Assay Substrate and Nano-Glo^®^ Luciferase Assay buffer (dilution: 1:50) to cells, and incubated for up to 10 min at 37 °C and 5% CO_2_ until cells were detached. Subsequently, luciferase expression levels were quantified using a Tecan ELISA reader.

### 2.3. Statistical Analysis

Statistical analyses were performed using GraphPad Prism version 8.3.0 for Windows, GraphPad Software, La Jolla California USA, www.graphpad.com. Data were presented as mean and standard error of the mean (SEM) or as absolute values with percentages or fold changes (*n* = 3 to 5). Comparisons between two groups were performed with unpaired/paired, two-tailed, and *t*-tests. Comparisons between more than two groups were performed with One-Way-ANOVA and Holm-Sidak’s multiple comparison posttest. The significance levels were set at * *p* < 0.05, ** *p* < 0.01, and *** *p* < 0.001.

## 3. Results

### 3.1. The Characterization of NAEPCs

**Figure 1:**
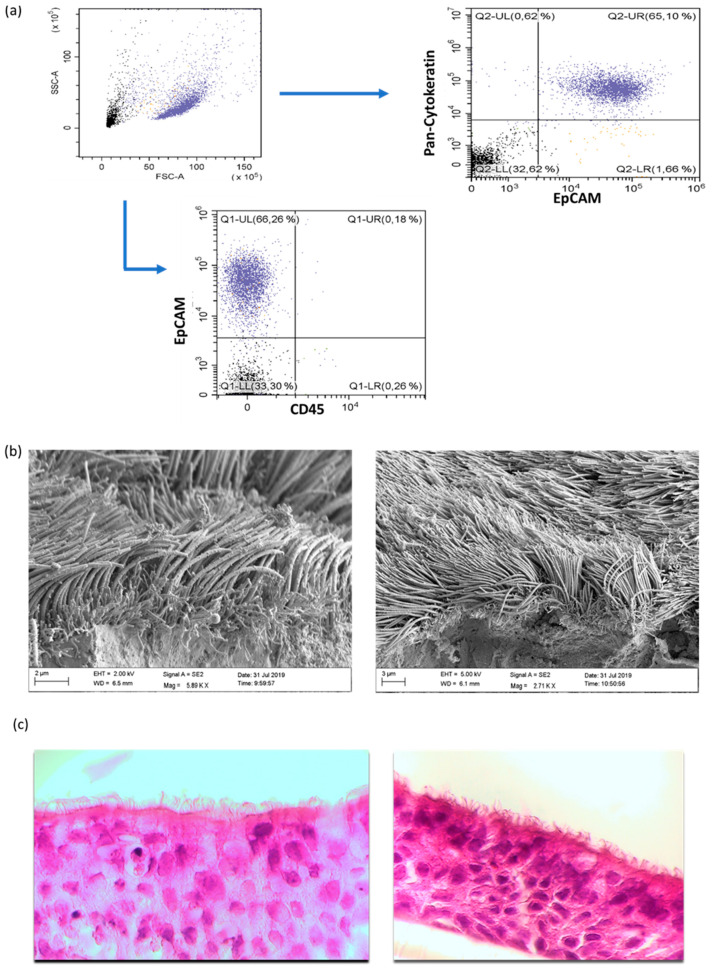
Characterization of primary human nasal epithelial cells (NAEPCs) derived from the pediatric exacerbation study cohort. (**a**) NAEPCs were obtained through nasal brushing procedure. After different washing and lysing steps, NAEPCs were seeded in collagen I pre-coated T75 flasks and cultured for up to 2 weeks until 80–90% confluency was reached. During the splitting process from passage 0 (P0) to passage 1 (P1), NAEPCs were harvested for flow cytometric analyses. Cells were stained with CD45-APC, CD326(EpCAM)-PE as surface antibodies. After fixation and permeabilization, anti-cytokeratin-FITC was used for intracellular staining. (**b**) After passage 2 (P2), the cells were seeded for organotypic 3D air–liquid interface cultures. Nasal mucus secretion and ciliary beats were observed through light microscopy after 6 to 8 weeks of culturing. Final specimens were then processed for raster electron microscopic imaging. (**c**) Histologic analyses confirmed the morphology of the nasal epithelial cell population (10× magnification).

To prove the purity of the cultured cells derived from our pediatric exacerbation study cohort, flow cytometric analyses were performed for each culture. As presented in Figure 1a, the isolated cells were CD45^neg^EpCAM^pos^Pan-Cytokeratin^pos^. In addition, the morphology of the cultured cells was analyzed through raster electron microscopy (REM) as well as histology, which revealed the purity of the cultures (Figure 1b,c).

### 3.2. Adenovirus Transduction Rates of NAEPC

**Figure 2:**
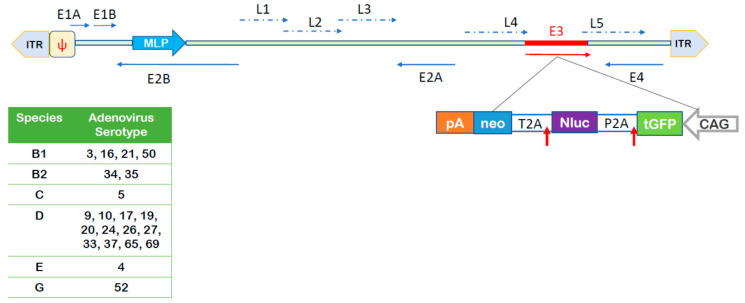
Summary of utilized adenoviruses (AdV) and schematic outline of DNA sequences contained in the AdV genomes. The upper panel provides a schematic overview of contained DNA sequences in the AdV genomes and the lower panel summarizes AdV types used for this study and respective species. The GFP, luciferase, neomycin (GLN)-reporter cassette encoding turboGFP (tGFP), nanoLuciferase (nLuc), and neomycin (neo) was inserted into the E3 region in the opposite orientation of major later promoter (MLP) of the adenovirus genome. ITR: inverted terminal repeat; Ψ: packaging signal; E1A, E1B, E2A, E2B, E3, E4: early adenovirus transcription units; L1-L4: late adenovirus genes, T2A and P2A: self-cleaving peptides; CAG: promoter with cytomegalovirus early enhancer element, part of the chicken beta-actin promoter and splice acceptor of the rabbit beta-globin gene; pA: polyadenylation signal. Blue horizontal arrows: early gene transcription units, blue dotted arrows: late gene transcription units, vertical red arrow: schematically presenting cleavage peptides. The figure was adapted from Zhang et al. [19].

To address the question whether NAEPCs were submissive to AdV infection, we transduced cultured NAEPCs in a monolayer with 21 different luciferase and GFP expressing AdV types (Figure 2) at 50 vpc. Twenty-four hours post-transduction, the luciferase activity of infected cells was determined.

**Figure 3:**
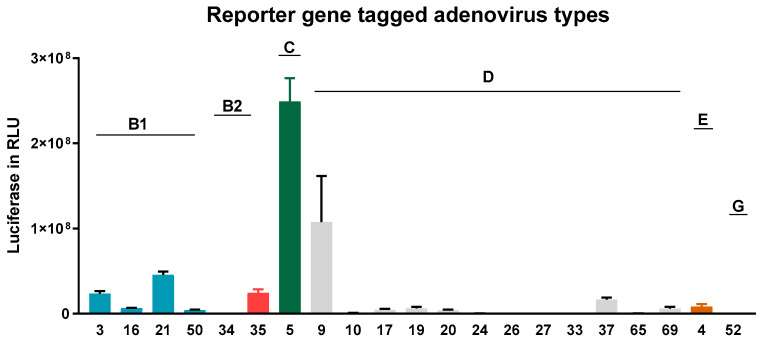
Adenovirus transduction efficiency in NAEPCs. The NAEPCs derived from healthy controls were infected with reporter gene tagged AdV types (*n* = 21) using 50 virus particles per cell (vpc). Twenty-five hours post-transduction, luciferase assays were performed. Several AdV types efficiently transduced NAEPCs. Infection with respiratory AdV types such as 3, 9, 16, 21, 50, and 5 revealed high transduction rates. Values were given as absolute numbers and presented as mean and standard error of mean (SEM) (*n* = 3).

**Figure 4:**
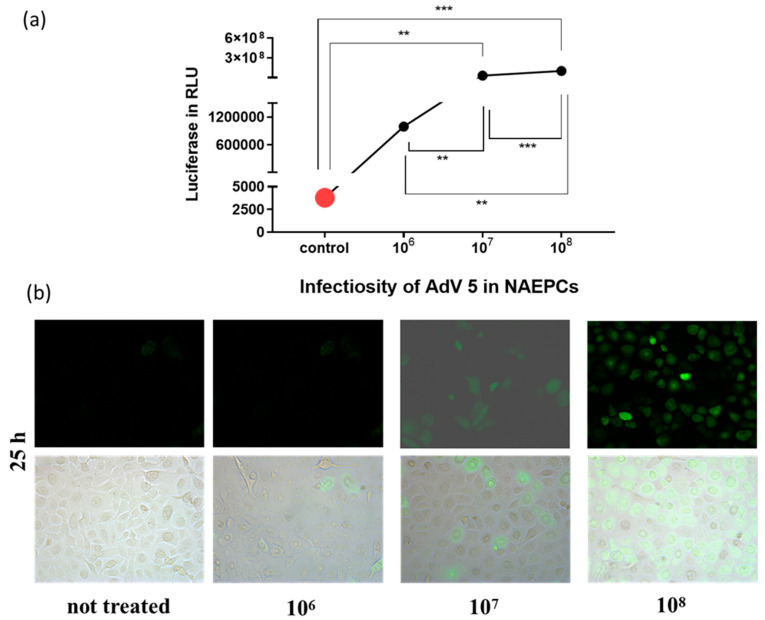
Evaluation of AdV5 infection in NAEPCs. The cells obtained from healthy controls were infected using different virus concentrations (1 × 10^6^, 1 × 10^7^, and 1 × 10^8^ vpc). Twenty-five hours post-transduction, NAEPCs were analyzed through luciferase assay and immunofluorescence microscopy. As presented in (**a**), the higher the AdV5 concentration, the higher the transduction efficiency in NAEPCs. (**b**) These results were confirmed by immunofluorescence microscopic analyses (10× magnification) (*n* = 3).

As shown in Figure 3, AdV type 5, followed by 9, 21, 3, and 35, showed the highest transduction rates in NAEPCs if directly compared to other AdV types. Therefore, AdV5, as a common respiratory virus and the most analyzed virus in terms of gene therapeutic approaches, was then used for further analyses. To determine the infections rates of AdV5 in NAEPCs, we transduced NAEPCs with AdV5 using different vpc and measured the luciferase expression level and visualized GFP^pos^ cells through immunofluorescence microscopy. Twenty-five hours post-transduction, there was a clear correlation between the virus dose, the luciferase expression levels, and the GFP^+^-expressing cell numbers. A cytopathic effect of infected cells associated with adenovirus infection was not observed (Figure 4a,b).

### 3.3. Stimulation with HDM Increased the Expression of AdV Specific Receptors

**Figure 5:**
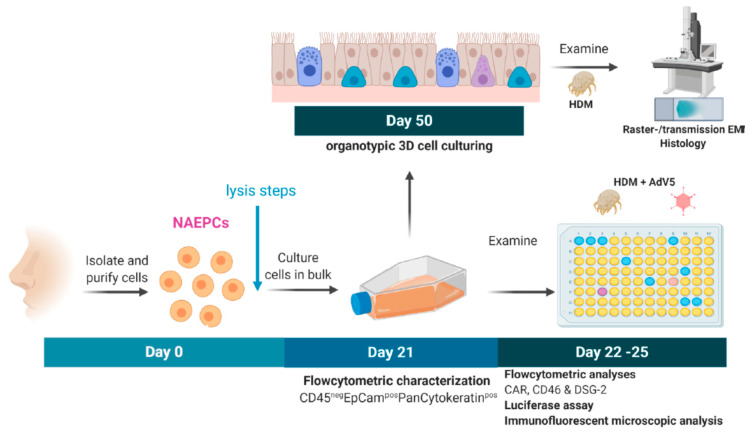
Experimental setup to analyze the effect of AdV and the house dust mite (HDM) on NAEPCs. During the nasal brushing procedure, we collected the cells in cell culture medium, centrifuged at 350× *g* for 8 min, and washed with PBS. The cell pellet was resuspended in cell culture medium, and the cells were seeded in collagen I pre-coated T75 flasks. After passage 2 (P2) was reached, NAEPCs were collected and seeded in collagen I pre-coated 96 well plates. Different HDM concentrations were used (1 µL/mL, 10 µg/mL, 100 µg/mL). The transduction concentration of AdV5 was set at 10 virus particle per cell (vpc). This figure was generated using BioRender.com

It is assumed that patients with allergies and asthma suffer from increased virus infections [22]. Therefore, we studied the virus transduction efficiency of AdV5 in HDM-provoked NAEPCs. Different concentrations of HDM (1 µg/mL, 10 µg/mL, and 100 µg/mL) were used to provoke NAEPCs ex vivo, and the schematic outline of this experiment is shown in Figure 5.

**Figure 6:**
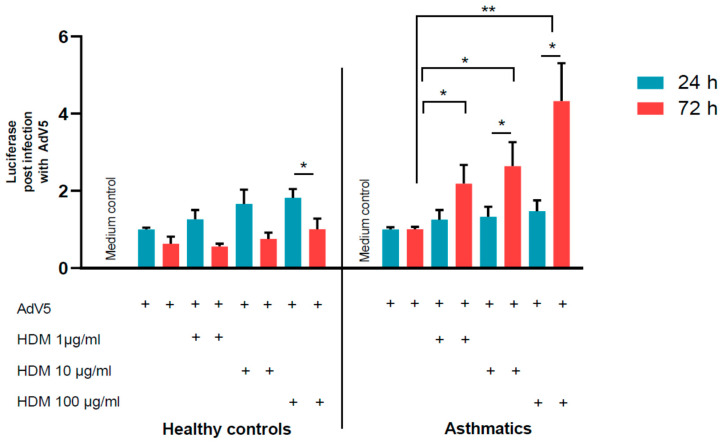
The effect of HDM stimulation and AdV5 infection on NAEPCs. NAEPCs were stimulated at different time points (day 1 and day 3) with different HDM concentrations (1 µg/mL, 10 µg/mL, and 100 µg/mL), and the cells were subsequently transduced with AdV5 at 10 virus particle numbers per cell (vpc). Twenty-four hours post-transduction, luciferase assays were performed. We observed an increased AdV5 transduction efficiency in pre-stimulated NAEPCs with HDM, particularly at day 3 in asthmatic specimens. This was in contrast to samples of healthy controls. Values were normalized and presented as fold change given as mean and standard error of mean (SEM).

Luciferase assays were performed one and three days post-transduction correlating with AdV transduciton rates. There was a trend of increased AdV transduction in healthy control cells and in cells derived from asthmatics. Especially on day 3 after HDM exposure, we observed an enhanced AdV mediated luciferase activity in HDM-provoked NAEPCs from asthmatics, compared to NAEPCs derived from healthy cells (Figure 6).

**Figure 7:**
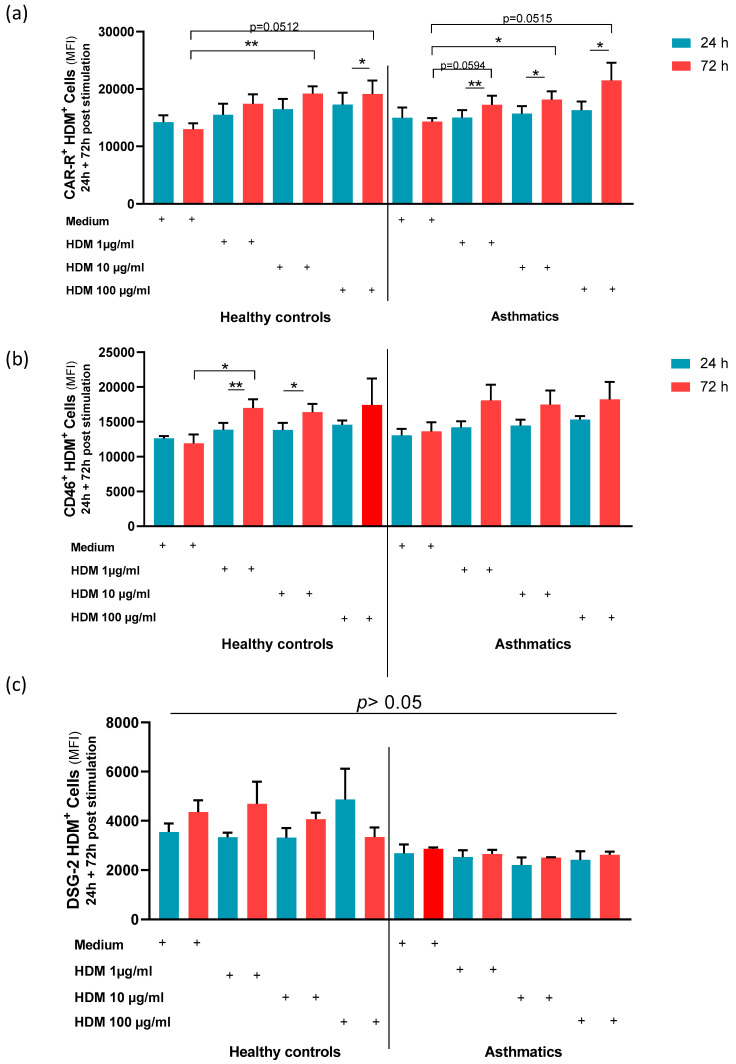
The effect of HDM on AdV receptors in NAEPCs. NAEPCs were stimulated with different HDM concentrations (1 µg /mL, 10 µg/mL, and 100 µg/mL) at different time points (days 1 and 3), and the coxsackie and adenovirus receptor (CAR), CD46, and Desmoglein-2 receptors expression levels were characterized by flow cytometry. (**a**) The mean-PE values for CAR were set as absolute numbers. As shown, the asthmatic group had significant differences at CAR expression levels after HDM stimulation, particularly at day 3 compared to day 1. (**b**) The mean-APC values for CD46 were set as absolute numbers. As shown, the healthy control group showed significant differences between time points and concentration levels, compared to the asthmatic group (**c**) Desmoglein-2-PE was not significantly different in terms of time points and concentration levels in healthy controls and asthmatics.

To shed light on the underlying mechanism for enhanced AdV5 uptake, major AdV receptor expression levels, in particular, CAR, CD46, and DSG-2, were characterized on NAEPCs through flow cytometry. In comparison to HDM-provoked NAEPCs, we observed a significant increase of CAR and CD46 expression levels on NAEPCs of asthmatics as well as on NAEPCs of healthy donors. Thus, we speculate that an increased AdV receptor expression level may explain the enhanced AdV5 transduction efficiency of NAEPCs after HDM exposition (Figure 7a,b). In contrast, there was no significant difference in the expression level of DSG-2 on HDM-treated NAEPCs, compared to healthy NAEPCs controls (Figure 7c).

### 3.4. HDM Increased the Permeability between Cell–Cell Contacts through Affecting the Epithelial Barrier Integrity

**Figure 8:**
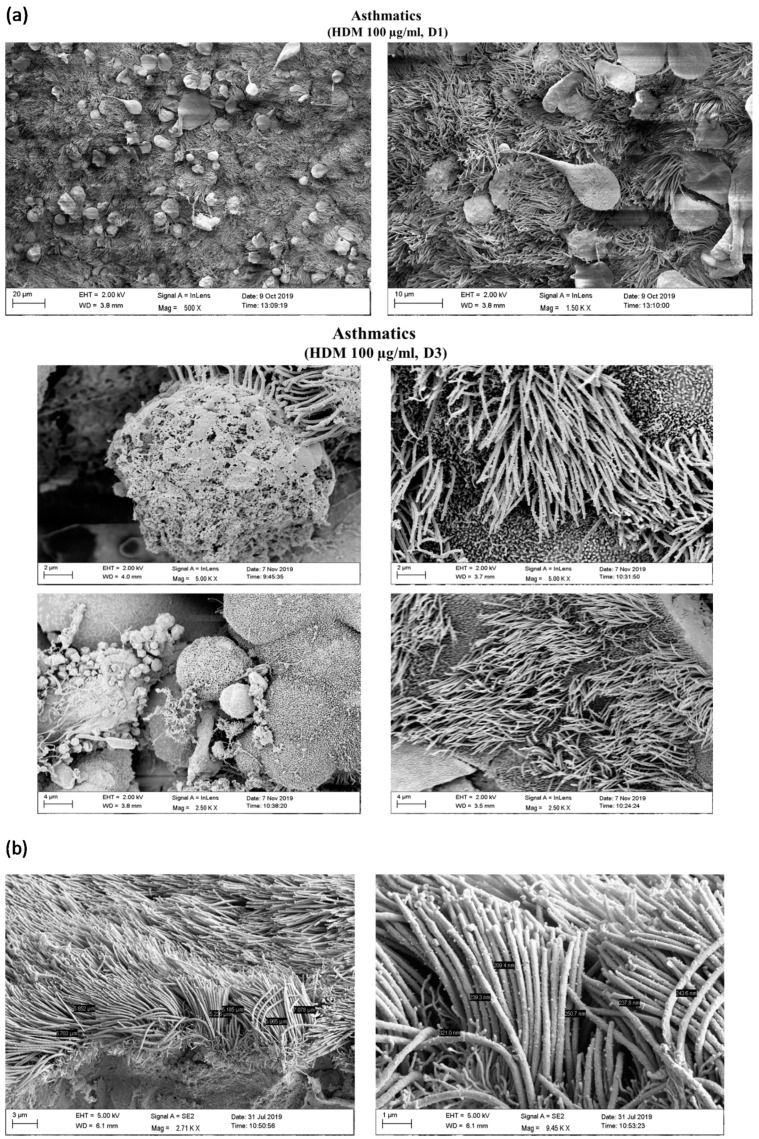

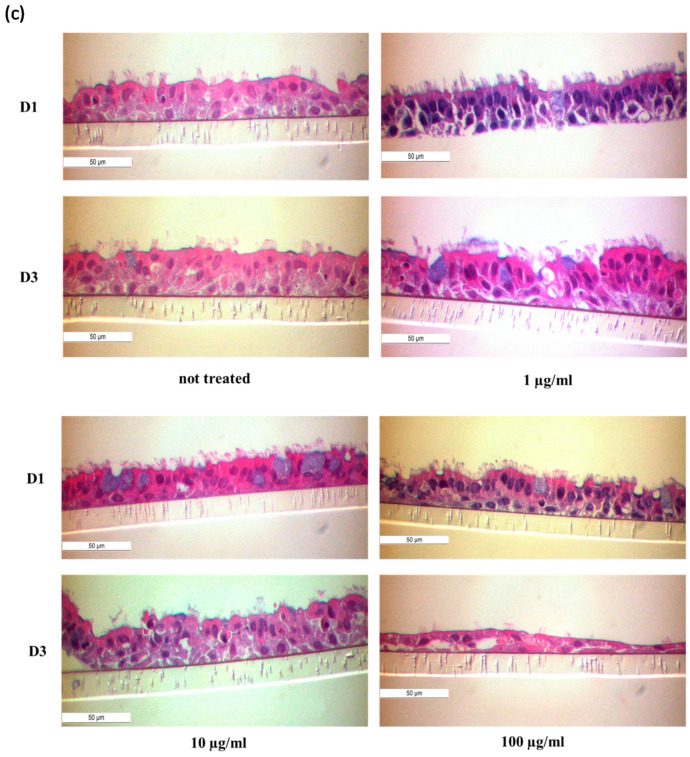

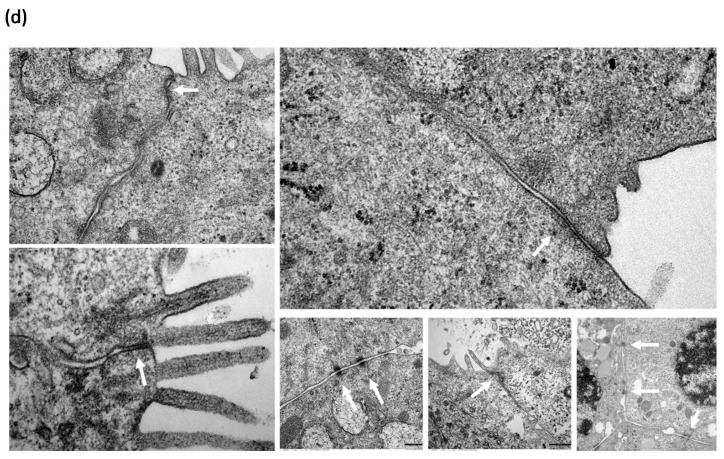
Characterization of NAEPCs in organotypic 3D air–liquid interface (ALI) cell cultures by electron microscopic imaging. (**a**) NAEPCs were used for 3D ALI cell cultures, were treated with HDM using different concentrations indicating time points, and were imaged with raster-electron microscopy. An irritated epithelium after HDM stimulation with 100 µg/mL was observed. Some cells were also detached from the cell population in the specimens of the asthmatic group. After day 3, we observed an irritation of the epithelium and an affected barrier integrity in some regions of the cultures (scale bars were added to the respective figures, different magnifications were used (500-fold to 2500-fold)). (**b**) Measuring the thickness of the cilia, there was a lack of significant correlations between treated and untreated, healthy, or asthmatic samples (scale bars were added to the respective figures, different magnifications were used (500× to 2500×)). (**c**) Histologically, the epithelium was affected and numerous mucus cells were observed when treated with high dose HDM (10× magnification). (**d,e**) Transmission electron microscopic analyses of untreated healthy (**d**) and asthmatic (**e**) 3D cultures revealed a different tight junction conformation, especially for asthmatic 3D cultures. The tight junctions in untreated asthmatic samples (**e**) were tightly packed and were present in a higher numbers compared to untreated control samples (**d**). The white arrows mark the respective tight junctions. Different magnifications were applied (20,000-fold to 50,000-fold).

To analyze whether HDM influences the barrier function on NAEPCs that may lead to enhanced AdV transduction, we provoked 3D NAEPCs cultures with HDM and performed raster-EM on days 1 (d1) and day 3 (d3). Raster-EM scan provided interesting insights into the irritated epithelium after high dosage of HDM, in particular at d3 (Figure 8a). Measuring the lengths and thickness of the ciliary, there was no statistical differences for the different groups (Figure 8b). Histologic analyses confirmed an increased mucus production and an irritated basal layer after HDM treatment (Figure 8c). Furthermore, we observed a different tight junction conformation in organotypic 3D NAEPCs cultures of asthmatics compared to healthy controls. The tight junction conformation in untreated asthmatic samples was tightly packed and was presented in a higher number than the untreated control samples (Figure 8d,e).

## 4. Discussion

Here, we characterized the effects of HDM exposition on AdV infection in an ex vivo cell culture models of NAEPCs of exacerbated pediatric asthmatics and healthy controls from our pediatric exacerbation study. With this experimental approach, we successfully characterized the effects of HDM exposition on human AdV infection in NAEPCs ex vivo.

Using a library of 21 luciferase- and GFP-tagged AdVs, we analyzed AdV infection rates in NAECPs in the presence of HDM as a common allergen. We found that AdV luciferase expression levels were significantly increased 3 days after HDM exposure in asthmatics, compared to healthy controls, hinting towards the hypothesis that replication of AdV may be influenced by HDM stimulation. However, this hypothesis needs to be further analyzed by performing AdV genome replication studies in NAECPs.

It was described previously that virus infection can synergize with allergens in the induction of asthma exacerbations. The first study describing the interaction between RV infection and HDM exposure was performed by Bossios et al. (2008) [23]. They observed an increase in cytokine levels in immortalized human bronchial epithelial cell line when RV and HDM were applied or when cells were provoked with HDM before RV infection [23]. Interestingly, Akbarshahi et al. (2018) found a similar effect by showing that HDM exposure affected the antiviral response provoked by virus infections [12]. Furthermore, Golebski et al. (2014) described a relationship between HDM and Poly I:C stimulation in terms of gene and protein expression [24]. The results of our study broaden the preceding findings, as we have observed an increased uptake of AdV5 after HDM stimulation potentially mediated by enhanced major AdV receptor expression. Our results represent the first in vitro evidence that HDM stimulation may increase susceptibility to AdV infections.

Adenovirus receptors, including CAR, CD46, or DSG-2, are required for uptake of AdV into the respective target cell. AdV5 mostly interacts with CAR, whereas CD46 and DSG-2 receptors are used by AdV3, 7, 14, and others. CAR is localized on the basolateral and apical surface of the epithelial cells. Lung epithelial cells are interconnected by tight junctions. By this formation, the basolateraly-located CAR receptors may protect the cell from the interaction with AdV [25,26,27]. The flow cytometric analyses of HDM-provoked NAEPCs showed higher levels of CAR on NAEPCs. In contrast to the work of Excoffon et al., our data do not include the analyses of a CAR localization shift but include the adenoviral susceptibility of HDM-provoked cells [27]. We speculate that an upregulation of AdV receptors upon HDM inhalation may lead to increased susceptibility to AdV infection in patients with allergic rhinitis or asthma exacerbation in vivo. However, to support this hypothesis, additional in vivo experiments are needed. We furthermore noticed a low but measurable increase of CD46 receptor expression levels on NAEPCs after HDM exposition. Contrary to our results, Tsai and colleagues (2018) observed decreased CD46 levels and increased apoptosis in primary nasal mucosa samples from adult mild asthmatics when cultured with HDM extracts [28].

NAEPCs gained increased attention as a model to study asthma development, to study immune responses during virus infection in asthma patients, and to define biomarkers specific for asthma and virus infections [29,30,31]. Their innate immune reactions such as Toll-like receptor pathways, secretion of IL-33, or Thymic stromal lymphoprotein boost adaptive immune responses and start a broad range of stimulation processes, e.g., activation of naïve T cells [18]. By using NAEPCs from healthy and allergic subjects, Vroling et al. showed that both groups had differences in the chemokine, growth factor, and transcription factor levels [16,17]. Thus, NAECPs represent a valuable alternative as an ex vivo model, compared to tracheal or bronchial epithelium of the small airways. This further supports the use of NAECPs in our study to explore the relationship between asthma development, HDM exposure, and AdV infections. Future studies should analyze immune responses in these cells after HDM exposure and AdV infection. Note that the used set-up of the present study was completely based on a primary human biomaterial database. This is in contrast to other published studies, which exemplarily used cancer cell lines to correlate their experimental findings with clinical observations.

In summary, this work provides novel insights into the mechanism of adenoviruses on the airway epithelium of asthmatics. To the best of our knowledge, this is the first ex vivo study presenting the impact of HDM sensitization on AdV infection in an in vitro exposition model. Our study may open new paths for the interaction between allergens and virus infections, and may provide a basis for further potential gene therapeutic approaches in treatment of childhood asthma exacerbation.

## 5. Conclusions

This work analyzed the interaction of the HDM exposition in the presence of AdV infection in an in vitro model based on NAEPCs. We demonstrate that a pre-stimulation with HDM may induce an increased AdV infection rate at least partially mediated by increased CAR receptor expression. Moreover, electron microscopy and histologic imaging revealed an effect on the cilia in organotypic 3D cell cultures when exposed to HDM that may be associated with an increased adenoviral transduction. To the best of our knowledge, this pilot work contributes novel aspects to the hypotheses regarding whether an allergic predisposition may increase an adenovirus infection.
